# Central American Mangrove Blue Carbon: Distribution, Dynamics and Future Directions

**DOI:** 10.1007/s13157-025-02025-4

**Published:** 2026-01-19

**Authors:** Nicholas T. Girkin, Steven W. J. Canty, Andre S. Rovai, Hannah K. Morrissette, Rachel Collin, Yashvini Shukla, Tania E. Romero-González, Jose Quirós, Jorge Pineda, Jacklyn Rivera Wong, Miguel Cifuentes-Jara

**Affiliations:** 1 School of Biosciences, University of Nottingham, Nottingham, LE12 5RD UK; 2 Smithsonian Environmental Research Center, Edgewater, MD 21037 USA; 3 Smithsonian Tropical Research Institute, Balboa, Ancon, Panama; 4 Conservation International, Arlington, VA 22202 USA; 5 Department of Oceanography and Coastal Sciences, Louisiana State University, Baton Rouge, LA 70803 USA; 6 National Biodiversity Conservation Commission, Ministry of the Environment, San José, Costa Rica

**Keywords:** Nature-based solution, Climate change adaptation, Climate change mitigation, Nationally determined contributions, Resilience

## Abstract

Mangroves are one of the most important Blue Carbon ecosystems within the tropics and subtropics, capturing and storing more atmospheric carbon dioxide per unit area than terrestrial forest systems. There is large variation in estimates of carbon stocks of mangroves in Central America, due to differences in underlying geomorphology, localised environmental conditions, and species composition, which also vary between the Pacific and Atlantic coastlines. In this review, we assess our current knowledge of the distribution of mangroves in the region, their role as a carbon sink, our current understanding of their dynamics and resilience, and identify key questions to understand their likely responses to future environmental change processes. This is of particular concern as the Central American region is predicted to experience significant climatic changes, such as increased air and sea surface temperatures, greater frequencies of high intensity cyclones, and increased drought events. Understanding the resilience and vulnerability of these systems will have policy and management implications for the role of Blue Carbon ecosystems as natural climate solutions, ecosystem-based adaptation plans, and disaster risk reduction strategies.

## Resumen

Los manglares son uno de los ecosistemas de carbono azul más importantes de los trópicos y subtrópicos, ya que capturan y almacenan más dióxido de carbono atmosférico por unidad de superficie que las marismas, las praderas marinas y los sistemas forestales terrestres. Existe una gran variación en las estimaciones de las reservas de carbono de los manglares de América Central, debido a las diferencias en la geomorfología subyacente, las condiciones ambientales locales y la composición de las especies, que varían a lo largo y entre las costas del Pacífico y del Atlántico. En esta revisión, se evalúo el conocimiento actual de la distribución de los manglares en la región, su papel como sumidero de carbono, la comprensión actual de su dinámica y resiliencia, se identificaron preguntas clave para entender sus probables respuestas a futuros procesos de cambio ambiental. Esto es de particular interés ya que se prevé que la región Centroamericana experimente cambios climáticos significativos, como el aumento de las temperaturas del aire y de la superficie del mar, una mayor frecuencia de ciclones de alta intensidad y un aumento de los eventos de sequía. Comprender la resiliencia y vulnerabilidad de estos sistemas tendrá implicaciones políticas y de gestión para el papel de los ecosistemas de carbono azul como soluciones climáticas naturales, planes de adaptación basados en ecosistemas y estrategias de reducción del riesgo de desastres.

## Introduction

Mangroves are tropical and subtropical ecosystems, and are amongst the most carbon dense ecosystems on Earth (Donato et al. 2011). Their capture and long term storage of atmospheric carbon dioxide, termed Blue Carbon, makes mangroves a crucial natural climate solution (NCS) (Nellemann 2009). Beyond carbon sequestration, mangroves provide several critical ecosystem services, ranging from their role as a significant reservoir of biodiversity (Ellison et al. 1999), to coastal defence (Menéndez et al. 2020), provisioning (including food security and fisheries support) (Akram et a., 2023), and habitat for permanent and seasonal bird rookeries (Lefebvre and Poulin 1996; McFadden et al. 2016). Further, mangroves have high cultural value for recreation, tourism, and education (Dahdouh-Guebas et al. 2021; Moore et al. 2022), with multiple different uses documented from pre-Columbian times to the present (López-Angarita et al. 2016). These cultural values vary across latitudinal and longitudinal scales, and in part are shaped by the geo-political differences among countries and Indigenous peoples (Canty et al. 2018; López-Angarita et al. 2016). Central America, a region home to approximately 52 million people, accounts for 3.0–3.4% of global mangrove cover, which supports a range of biodiversity along both the Pacific and Atlantic coastlines, and provides an array of ecosystem services to millions of people in coastal communities. The Mesoamerican region more broadly (in particular Caribbean coastal zones of Yucatan Mexico, Belize, Guatemala, and Honduras) is a global hotspot of mangrove co-occurrence with coral reefs, and seagrasses (Carlson et al. 2021). This co-occurrence greatly enhances mangrove coastal resilience and protection capacities, and reduces the vulnerabilities of coastal communities (Carlson et al. 2021). Moreover, coral reef fisheries are enhanced where healthy reefs neighbour healthy mangrove ecosystems (Mumby et al. 2004), which together have the potential to support the livelihoods and food security of coastal communities (Canty et al. 2019; Canty and Deichmann 2022).

Despite their importance, like other tropical and subtropical wetlands (e.g. seagrasses), mangroves are under threat from land use and climate change impacts. Conversion of mangroves to agriculture and aquaculture remain some of the greatest threats to mangroves, even in protected areas (Castillo et al. 2021; Goldberg et al. 2020). Between 1960 and 2010, 20–35% of global mangrove extent was lost (Polidoro et al. 2010). Although deforestation rates have slowed and there is optimism due to increased actions to protect and restore mangrove ecosystems (Friess et al. 2019), mangrove degradation is ongoing, and the magnitude is largely unknown at local and global scales (Adame et al. 2024). In addition, losses in mangrove extent and increases in degradation are associated with varying levels of fragmentation, which can lead to a reduction in ecosystem functions such as their ability to trap and retain sediments (Thampanya et al. 2006), abate wave energy (Dahdouh-Guebas et al. 2005), and keep pace with sea-level rise (Schuerch et al. 2018). Ambitious conservation and restoration targets are required to both protect existing and rebuild lost mangrove ecosystems and their associated ecosystem services (Buelow et al. 2022). This could substantially enhance regional rates of carbon sequestration and resultant carbon stocks, if deployed at scale. Protected areas are one tool, but top down measures have different levels of effectiveness; more localized community-based management efforts are positively associated with gains in mangrove cover (Hagger et al., 2022).

Mangrove management can be complex as they can be considered marine or terrestrial ecosystems depending on the country, with management strategies varying considerably both at national and sub-national levels (López-Angarita et al. 2016; Canty et al. 2018). Additionally, mangrove forests may cross national and international boundaries, and the stakeholders and mangrove dependents in these areas utilise and value mangroves differently (Golebie et al. 2022). The valuation and perception of mangroves have significantly changed in the last few years, particularly by governments, due to the climate mitigation potential of mangroves and the potential of financial gains from trading Blue Carbon in voluntary and compliance carbon markets (VCMs and CCMs, respectively), in addition to their role of enhancing coastal resilience. VCM interest primarily comes from the private sector to credit actions in reducing greenhouse gas (GHG) emissions and is generally associated with reducing environmental footprints and enhancing the public image of a company (LeBlanc 2014). Within Central America there are at least two VCM Blue Carbon restoration projects being developed in Honduras (Friess et al. 2022), with more likely under development in the wider region, enhancing potential carbon stocks if realised at scale. Mangrove restoration and conservation can form part of a much wider series of ecosystem-based adaptation actions, focusing on enhancing resilience to climate hazards, for example through improved flood defence and coastal storm protection (Menéndez et al. 2020). This will become increasingly important under the intensifying impacts of climate change, as the Central American region faces greater frequency of high intensity hurricanes, with the degradation and loss of mangrove ecosystems increasing coastal communities’ vulnerability to these events (Amaral et al. 2023; Mo et al. 2023; del Valle et al. 2020).

There is a renewed need and interest in understanding differences in ecosystem dynamics of mangroves, to assess their distribution, and potential for climate change mitigation and adaptation, at local, national, regional, and global scales. This is increasingly important as countries utilise mangroves and other ecosystems as NCSs to deliver on their Paris Agreement commitments. Countries that ratify the agreement are required to create and adopt nationally determined contributions (NDCs), which describe their national GHG emission-reductions and climate adaptation commitments (UNFCCC 2023). Unique to each country’s resources and state of local environment, NDCs are updated every five years to monitor progress towards the self-determined targets, and to enhance ambitions. In the most recent NDC synthesis report summarizing NDC 2.0s, from 166 NDCs submitted, 151 countries have specifically included coastal wetlands as nature-based solutions, and three Central American countries (Belize, Costa Rica, and Panama) specifically mention mangroves (UNFCCC 2023). The heightened interest for greater management, protection, and restoration, generally through NDCs, make the Central American region an interesting case study for understanding mangrove distribution, carbon dynamics, and resilience (Table 1). In this review we build on previous knowledge of mangroves in the region which to date have largely focussed on conservation and restoration, national assessments (e.g. (Morrissette et al. 2023), or global assessments of distribution and dynamics (Rovai et al. 2018, 2021a, b), to synthesise existing knowledge on the distribution, dynamics, and carbon stocks of Central American mangroves, and identify key remaining research priorities for the region.

**Table 1:** Central American Paris agreement commitments, and the inclusion of mangroves in their nationally determined contribution (NDC)

Country	Paris Agreement Ratification	NDC ambitions	NDC Version	Reference
Belize	4 November 2016	Protect an additional 14,000 ha and restore 4,500 ha by 2035.	3.0	Government of Belize, 2025
Guatemala	24 February 2017	Restore 1,500 ha mangrove by 2025, in participation with local and Indigenous communities, and the Garifuna.	2.0	Government of Guatemala, 2021
Honduras	4 November 2016	Updated mangrove emissions factors through the national forest inventory; inclusion in broader targets for forest restoration	2.0	Government of Honduras, 2025
El Salvador	26 April 2017	Establish the conservation and sustainable management of mangroves as a national priority; target restoration of 2,000 ha	2.0	Government of El Salvador, 2021
Nicaragua	22 November 2017	Reduce CO_2_ emissions generated by gross deforestation (including mangroves) at the national level by 25% by 2030 relative to the national baseline	2.0	Government of Nicaragua, 2025
Costa Rica	12 November 2016	Conserve and protect all coastal wetlands included in the National Registry of Wetlands; an aspiration to stop and/or revert the loss of coastal wetlands by 2030, focusing on addressing main causes deforestation of degradation that pose a threat to their health; explore the potential of public-private partnerships to advance mangrove protection and restoration efforts	1.0	Government of Costa Rica, 2020
Panama	4 November 2016	Increase mangrove cover by 1,800 ha by 2028 Include at least 50% of all mangroves into SINAP by 2026 Improve effective management of mangroves by 2027	3.0	Government of Panama, 2025

## Mangrove Distribution and Species Composition in Central America

Central America includes a substantial karst landscape, covering approximately 39,300 km^2^ which encompasses northeastern Guatemala, Belize, Honduras, and Nicaragua, as well as smaller areas of other Central American countries, which can favour mangrove formation (Kueny and Day 2002). Data on resulting mangrove distribution can be found from studies at local, national, regional, and global scales (Table 2), with mangroves distributed through the over 6,500 km of coastline in the region. Recent studies across Central America include assessments in Belize (Cissell et al. 2021) and Panama (Viquez et al. 2025). Mapping efforts using higher resolution imagery at the national level can estimate greater mangrove extent associated with improved mapping of mangrove cays and small areas of fringe mangrove (e.g. Cissell et al. 2021), although comparisons across studies are complicated by different workflows (Canty et al. 2025; Viquez et al. 2025). Regardless of the resolution and methodology most assessments broadly agree on the relative contributions of individual countries to total mangrove area. Combined, Central American mangroves account for 3.0–3.4% (or 4,411–4,960 km^2^) of the total global mangrove extent, estimated using synthetic aperture radar (SAR) (Bunting et al. 2022) and Sentinel 2 data (Jia et al. 2023), respectively (Table 2).

**Table 2:** Mangrove distribution in Central American countries. national area and total coastline length estimates are derived from CIA, 2023

Country	National area (km^2^)	Coast(s)	Total coastline length (km)	Mangrove area (km^2^)	Mangrove area relative to total land area (%)	Reference
Belize	22,966	Atlantic	386	302	1.31	(Hamilton and Casey 2016)
				529	2.30	(Bunting et al. 2022)
				579	2.52	(Cissell et al. 2021)
				337	1.46	(Jia et al. 2023)
Guatemala	108,889	Atlantic	400	253	0.23	(Hamilton and Casey 2016)
		Pacific		250	0.23	(Bunting et al. 2022)
				320	0.30	(Jia et al. 2023)
Honduras	112,492	Atlantic	832	524	0.47	(Hamilton and Casey 2016)
		Pacific		606	0.54	(Bunting et al. 2022)
				873	0.78	(Jia et al. 2023)
El Salvador	21,041	Pacific	307	236	1.12	(Hamilton and Casey 2016)
				373	1.77	(Bunting et al. 2022)
				378	1.80	(Jia et al. 2023)
Nicaragua	130,373	Atlantic Pacific	910	552	0.42	(Hamilton and Casey 2016)
				747	0.57	(Bunting et al. 2022)
				941	0.72	(Jia et al. 2023)
Costa Rica	51,100	Atlantic	1,290	506–528	1.03–0.99	(SINAC 2015)
		Pacific		325	0.64	(Hamilton and Casey 2016)
				371	0.73	(Bunting et al. 2022)
				413	0.81	(Jia et al. 2023)
Panama	75,517	Atlantic	2,490	1,328	1.75	(Hamilton and Casey 2016)
		Pacific		1,536	2.03	(Bunting et al. 2022)
				1,699	2.25	(Jia et al. 2023)
				1,837	2.27	(Viquez et al. 2025)
				1,871	2.48	(Prensa 2021)

In the region, Panama is the country with the largest mangrove area (1,536 km^2^), ranked 25th globally, followed by Nicaragua and Honduras, at 747 km^2^ and 129 km^2^, respectively (Bunting et al. 2022). The region is home to some of the tallest mangrove trees in the world; globally, Panama ranks 4th, Costa Rica 5th, and El Salvador 11th (Simard et al. 2019). Furthermore, the carbonate coasts of Bocas del Toro along Panama’s Caribbean coast and Belize are home to extensive mangrove cover which allocate most carbon to below-ground biomass in response to nutrient limiting conditions common of coastal karstic landforms (Rovai et al. 2018). Understanding the spatial distribution of mangroves, and their ecotypes, is essential for informing management decisions made at local to national scales, including directing conservation and restoration activities. However, unsurprisingly, differences in methodology and definitions result in a wide range of estimates of potential mangrove distribution (Bunting et al. 2022; Cissell et al. 2021; Friess 2023; Hamilton and Casey 2016; Jia et al. 2023). The definition of the mangrove ecosystem is important as this is the criterion that comprises the land use and land cover categories used in the mapping of these ecosystems. Therefore changes in these definitions can significantly affect mangrove cover estimates (Acosta-Velázquez et al. 2023), and the associated carbon stocks.

Across the region, there are seven true mangrove species belonging to four genera in Central America: *Rhizophora mangle and R. racemosa* (red mangrove), as well as the hybrid *Rhizophora x harrisonii*; *Avicennia germinans* and *A. bicolor* (black mangrove); *Laguncularia racemosa (*white mangrove); *Pelliciera rhizophorae* and *P. benthamii* (tea mangrove) (Saenger 2002). There are also a number of mangrove associated species throughout the region, including *Acrostichum aureum* (golden leather fern), *Conocarpus erectus* (buttonwood), and *Mora oleifera* (mora tree) (López-Angarita et al. 2016). Depending on the country, mangrove associated species may or may not be included in the official national definition of the mangrove ecosystem. For example in Guatemala, Panama, and Honduras, buttonwood is considered part of the mangrove ecosystem (Canty et al. 2018). Within the global context the Atlantic East Pacific biogeographic region, within which Central America is situated, is associated with the lowest mangrove diversity (Saenge, 2002). Along both Caribbean and Pacific coastlines mangroves can form monospecific zones, or feature dominance of a singular species, based on their tolerance to salinity and inundation. However, these nuances may not be evident in remote sensing, a methodology that is increasingly being applied for demarcating and monitoring mangrove cover.

## Mangrove Dynamics

### Contemporary Carbon Stocks Dynamics

Mangrove dynamics, including mortality and recruitment, ecosystem productivity, carbon burial, and vulnerability to anthropogenic change are strongly influenced by coastal geomorphology, and the wider landscape (Worthington et al. 2020). Geomorphological settings of Central American mangroves include deltas (e.g. the Térraba-Sierpe delta in Costa Rica), estuaries (e.g. Jaltepeque Bays in El Salvador), peatlands (e.g. San San Pond Sak in Panama), lagoons (e.g. Guaimoreto Lagoon in Honduras,), carbonates (e.g. in Belize), and open coasts across Central America (see Worthington et al. 2020 for a complete inventory of coastal geomorphic typologies across Central America). Geomorphological settings have been shown to exert strong influence on carbon stock allocation between above- and below-ground biomass and soil (Kauffman et al. 2020; Rovai et al. 2018, 2021a, b), and type of carbon stored (Arnaud et al. 2025). Above-ground biomass decreases from deltaic and macrotidal systems to lagoons to carbonate settings in response to reduced relative contribution of riverine nutrient loading (Rovai et al. 2021a, b). In contrast, per unit area soil organic carbon stocks increase with diminishing riverine inputs reflecting fertility gradients that control carbon allocation between roots and shoots (Twilley et al. 2018).

Sea level history is also a key factor at larger time scales as it creates both vertical and lateral accommodation space for particles settling and sequestration of both allochthonous and autochthonous carbon (Rogers et al. 2019). Carbon sequestration rates in mangrove soils are higher in river-dominated coastlines as a function of high deposition of river-borne mineral and organic particles, whereas it is lower in carbonate settings where most of the accumulated soil carbon is slowly produced in situ as a balance between plant productivity and decomposition (Breithaupt and Steinmuller 2022). Central America mangroves are subjected to both geomorphic and sea level forcings with micro-tidal regimes along the Caribbean and meso- or macro-tidal regimes along Pacific coast. Coastal typologies range from high rainfall river-dominated coastlines (Gulf of San Miguel, Darién (Suman 2007), and Osa Peninsula along Panama and Costa Rica’s Pacific coast, respectively, and nearly all along Central America’s Caribbean coast, to dry climate zones (e.g. Northern Costa Rica, Nicaragua, Honduras, El Salvador and Panama’s Arco Seco along the Pacific coast).

**Fig. 1:**
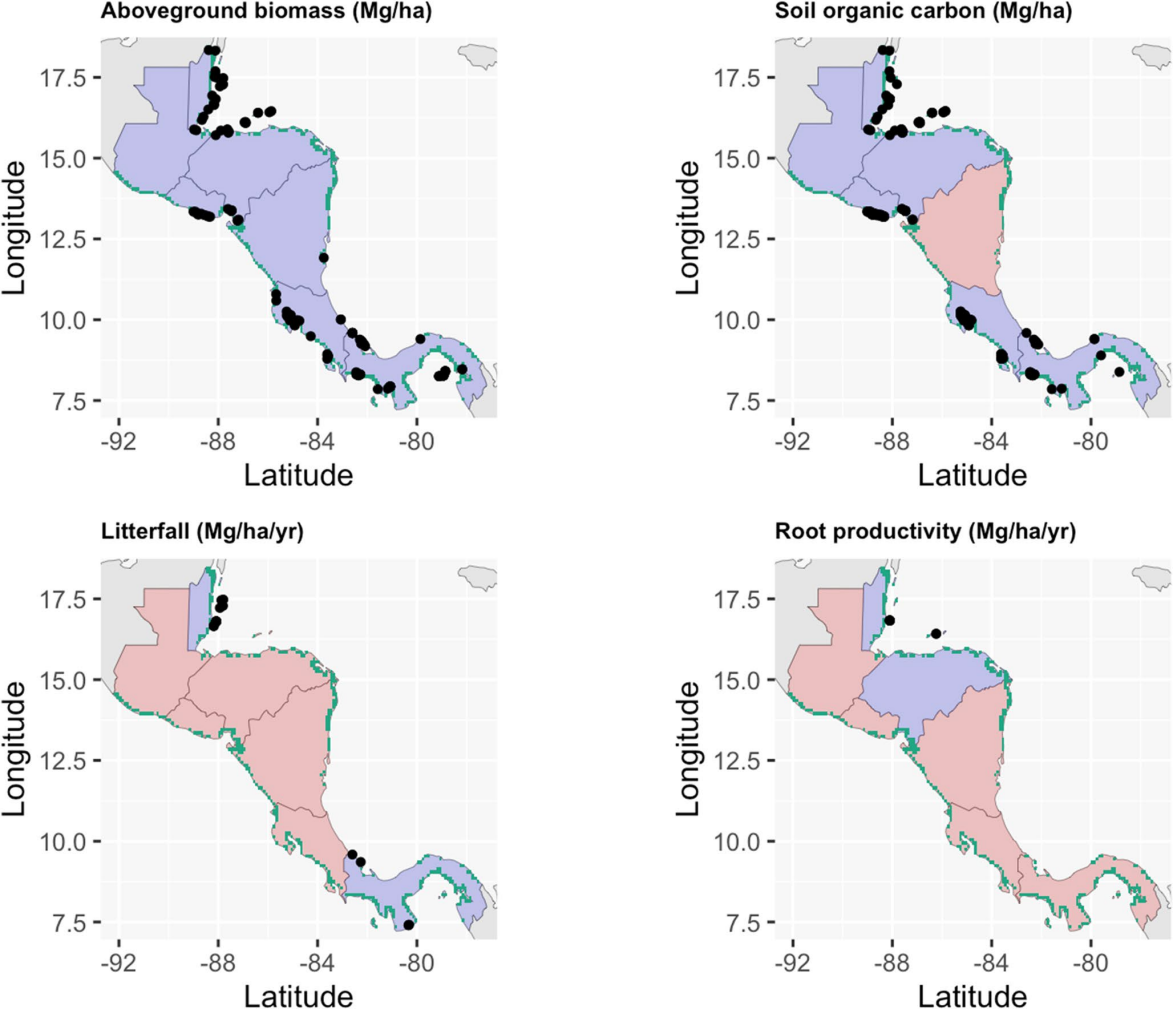
Distribution of accessible data on (**a**) above-ground biomass, (**b**) soil organic carbon stocks, (**c**) litterfall productivity, and (**d**) root productivity across Central America shown as black circles. Blue and red shading denote countries with and without data, respectively. Green shading shows the distribution of mangrove forests (Bunting et al. 2022). Above-ground biomass data from Rovai et al. 2021a, b, plus unpublished data (two sites in Panama and two sites in Guatemala); soil organic carbon stocks from the Coastal Carbon Atlas (available at: https://shiny.si.edu/coastal_carbon_atlas; (Holmquist et al. 2024); litterfall productivity from (Adame et al. 2024), and root productivity from (Arnaud et al. 2023). Little published data on soil organic carbon burial rates, greenhouse gas emissions, and lateral carbon exchange were found

Mangrove carbon sink capacity comprises above- and below-ground biomass alongside carbon storage in soils and sediments. Various recent studies have provided estimates of carbon storage for each component, including for Belize (Morrissette et al. 2023) (Fig. 1; Table 3**)**. Large above-ground biomass and carbon stocks, however, do not necessarily translate to increases in below-ground or soil/sediment carbon stocks (Kauffman et al. 2020). In certain locations (e.g. San San Pond Sak, Panama; and Gandoca, Costa Rica), mangroves coincide with the presence of peat (Phillips et al. 1997; Upton et al. 2018), high carbon organic matter comprising partially decomposed plant material that accumulates under acidic and anoxic conditions. Compared to the formation of peat from other tropical vegetation (e.g. hardwood and palm dominated swamps), little is known about the potential formation of mangrove-derived peat, despite its importance in determining below-ground carbon storage in coastal tropical wetlands. However, data from multiple sites supports the notion that these specific locations contribute significantly to the overall scale of mangrove Blue Carbon stocks. For example, differences between Honduran Pacific and Atlantic mangrove carbon stock are respectively 570 and ~ 1,000 Mg C ha^− 1^ (assessed to the bottom of mangrove peat, approximately 270 cm; Bhomia et al. 2016). Similarly, (Upton et al. 2018) reports potential carbon storage of 1,771 Mg C ha^− 1^ in the top 300 cm of soil in the San San Pond Sak wetland, Panama, where a fringe of mangrove overlies substantial peat deposits.

**Table 3:** National mangrove blue carbon stocks (Simard et al. 2019) and estimated yearly climate mitigation potential and value of financially viable Mangrove blue carbon (Zeng et al. 2021) for central American countries. Climate mitigation potential refers to additionality based on projected rates of mangrove loss (i.e. avoided emissions) in total national carbon pools (above-ground, below-ground, and soils). Net present value represents return on investment (i.e. profitability, based on costs of establishment, and nationally weighted-maintenance costs relative to national gross domestic product, and a constant carbon price of US$5 tCO_2e_ for the first 5 years and 5% appreciation over 30 years thereafter, and a 10% discount rate based on potential risk) of financially viable mangrove blue carbon

Country	Total carbon stock (Mg C)	Climate mitigation potential (tCO_2_e yr ^− 1^)	Net present value (US$ yr ^− 1^)
Belize	16,717,298	7,000 (± 3,000)	128,000 (± 86,000)
Guatemala	12,548,011	2,000 (± 0)	48,000 (± 17,000)
Honduras	21,857,392	38,000 (± 11,000)	1,286,000 (± 452,000)
El Salvador	11,216,253	16,000 (± 5,000)	561,000 (± 167,000)
Nicaragua	23,354,504	51,000 (± 14,000)	1,760,000 (± 645,000)
Costa Rica	13,998,83	10,000 (± 5,000)	211,000 (± 211,000)
Panama	58,979,743	45,000 (± 15,000)	796,000 (± 346,000)
Central America	**73**,**145**,**447**	**169**,**000**	**4**,**790**,**000**

**Fig. 2:**
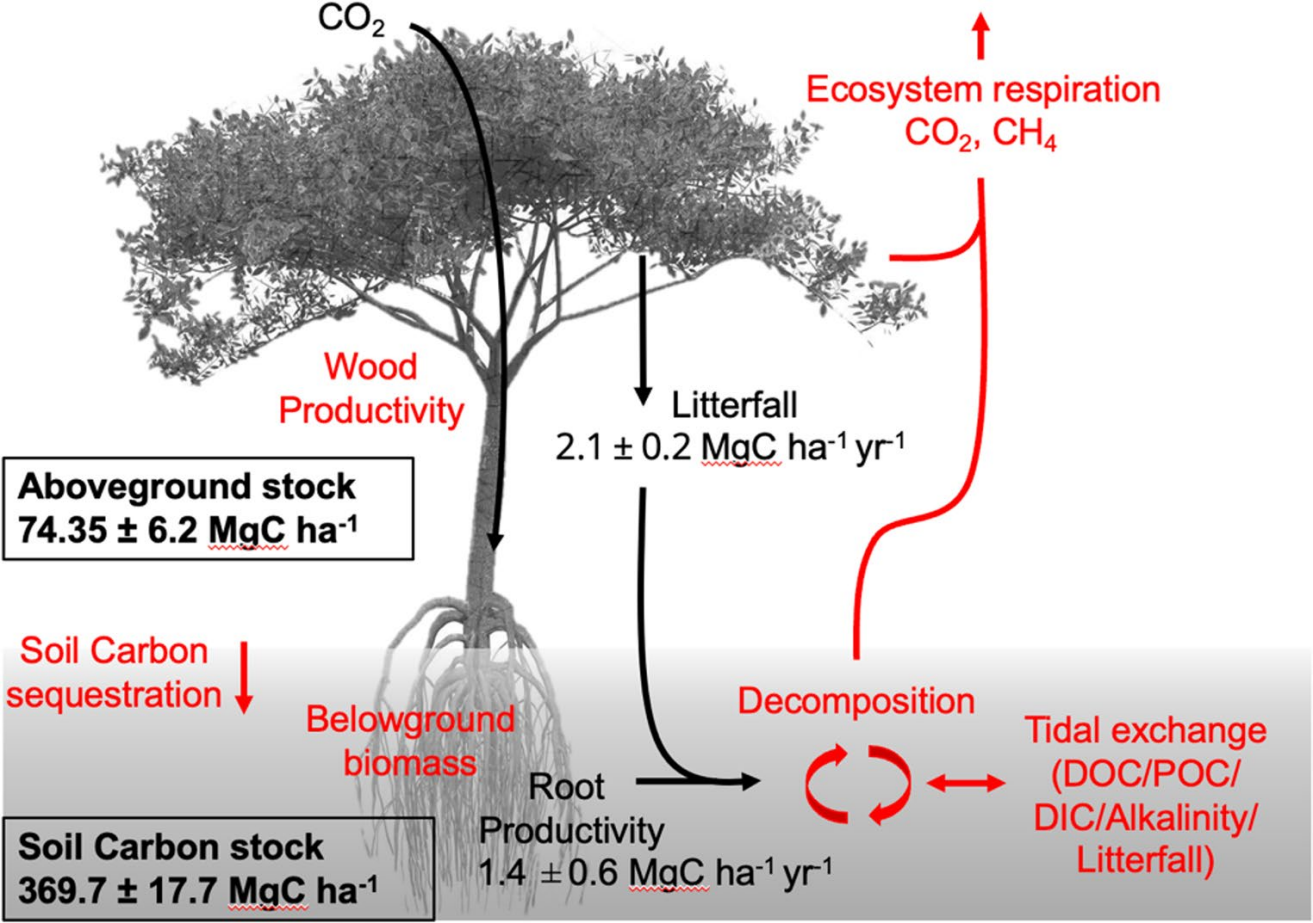
Existing (black font and arrows, estimates from Belize) and missing (red font and arrows) carbon stock and flux observations recorded for Central American mangroves. Above-ground and soil organic carbon stocks from (Morrissette et al. 2023), litterfall from (Koltes et al. 1998), and root production from (Mckee et al. 2007)

Net carbon sequestration in each pool is determined by several processes, including rates of plant productivity, litterfall, root turnover and exudation, and rates of loss through decomposition and lateral fluxes (Adame et al. 2024; Simpson et al. 2023) **(**Fig. 2). All of these can be impacted by anthropogenic change, including disturbances (e.g. oil spills), land use change, and climate impacts. Data on ecosystem productivity, turnover and losses, soil and vegetation CO_2_ and CH_4_ emissions, net tidal exchange (e.g. total alkalinity, dissolved organic and inorganic carbon, particulate organic carbon), and net sequestration are largely lacking for mangroves from across geomorphological settings from the Pacific and Caribbean coasts (Adame et al. 2024; Breithaupt and Steinmuller 2022). Global data for many processes are available, however, and highlight the important contribution of roots in determining soil carbon sequestration (Arnaud et al. 2023). Likewise, at local scales, the organic chemistry of the accumulated litter detritus that can determine the scale of the soil carbon sink is determined by species composition (Upton et al. 2018). Evidence from tropical peatland ecosystems in Central America (Girkin et al. 2019) has demonstrated that changes in peat organic chemistry (exemplified through ratios of aliphatic: aromatic carbon), can result in significant changes in decomposition rates for peat with increased proportions of labile carbon, accelerating the production of GHGs, including methane and carbon dioxide (Upton et al. 2018).

### Mangroves and Anthropogenic Change

The scale of the Central American Blue Carbon pool is only as important as its resilience to environmental change. Whilst mangroves are being championed as important NCSs, these ecosystems are highly vulnerable to anthropogenic and environmental change processes. Globally, 2.1% (3,363 km^2^) of mangrove cover was lost between 2000 and 2016, with an annual rate of loss of 0.13% (Hamilton and Casey 2016) (Table 4). Central American mangroves have long sustained anthropogenic activity, predating the pre-Columbian period (López-Angarita et al. 2016). However, these early uses of mangroves posed little threat given their relatively low intensity and dispersed nature of activities, matching similar trends reported for other tropical wetland ecosystems globally (e.g. tropical peatlands; Cole et al. 2022; Girkin et al. 2022). Major exploitation of the mangroves began under the Spanish, using material in construction and for shipbuilding (Lopez-Anguarita et al. 2016). The demand for mangrove wood was sufficiently high that the Spanish monarchy devised regulations to control its exploitation, including permits and licenses for certain species, or to cover certain areas (Lopez-Anguarita et al. 2016). Various anthropogenic activities in the 20th and early 21 st centuries further increased in intensity and geographic spread, resulting in extensive alterations and losses. However, in more recent years, that rate of loss has globally slowed, but key anthropogenic activities in Central American mangroves still impact ecosystem dynamics, including clearance for aquaculture, agriculture, timber extraction, coastal development, fluxes in hydrology and run-off as a result of dams and other land use changes (Hogarth 2015), oil spills (Duke et al. 1997), and overfishing.

**Table 4:** Net changes in mangrove distribution from (Bunting et al. 2022; Hamilton and Casey 2016)

Country	Study period	Total decline (%)	Rate of change (% per year)
Belize	1996–2020 2000–2012	3.70 1.12	0.15 0.09
Guatemala	1996–2020 2000–2012	0.14 6.41	0.01 0.53
Honduras	1996–2020 2000–2012	2.97 2.00	0.12 0.17
El Salvador	1996–2020 2000–2012	0.88 0.34	0.04 0.03
Nicaragua	1996–2020 2000–2012	2.06 0.74	0.09 0.06
Costa Rica	1996–2020 2000–2012	2.18 0.35	0.09 0.03
Panama	1996–2020 2000–2012	1.45 0.30	0.06 0.02
Central America	1996–2020 2000–2012	1.98 1.32	0.08 0.12

Throughout Central America the exact nature of threats differs both along and between Atlantic and Pacific coastlines. For example, within Panama Bay (Pacific coast of Panama) illegal mangrove deforestation and degradation for the development of infrastructure and urban expansion has been reported within protected areas (Castellanos-Galindo et al. 2017; Chamberland-Fontaine et al. 2022). Across Central America, agriculture is the dominant land use adjacent to mangroves and represents a significant driver of deforestation and degradation, as well as direct clearing of mangroves to install shrimp ponds on the Pacific coast (López-Angarita et al. 2016). Moreover, agriculture and aquaculture can indirectly affect mangrove ecosystem dynamics. For example, eutrophication and excessive nutrient loading can alter plant growth and function (Lovelock et al. 2006), which in turn impacts carbon budgets (Lovelock et al. 2015; Simpson et al. 2021), which is of concern within the Mesoamerican reef region where significant agriculture-based nitrogen pollution is moving through the watersheds (Berger et al. 2022). Degradation and loss of mangrove forests has been reported following oil spills (Duke et al. 1997), with potential legacy effects in sediments (Choudhury et al. 2021). Climate impacts that will affect mangrove ecosystem dynamics include sea level rise (Saintilan et al. 2023), alterations in the frequency and intensity of precipitation (Mafi-Gholami et al. 2020), cyclone events, increases in CO_2_ concentrations, rises in air and sea temperatures, and changes in salinity (Chowdhury et al. 2023). Mangroves can be highly sensitive to changes in salinity and the intensity and frequency of inundation, particularly when they exceed the tolerances of individual species (Daru et al. 2013), resulting in increased mortality and changes in species composition. This could be a major driver of change, with Central America and the Caribbean region already experiencing sea level rise of 6.15 ± 0.5 mm yr^− 1^ between 2004 and 2019, 67% faster than the IPCC’s global mean seal-level rise (3.69 ± 0.5 mm yr^− 1^; IPCC 2021; Maitland et al. 2024). Understanding if and where mangroves can keep pace with sea level rise via increased elevation driven by accretion, will determine future range distributions, especially where there is limited capacity to expand inland to higher ground (Schuerch et al. 2018) i.e. coastal squeeze. There remains a lack of clarity on land tenure, or urban or coastal infrastructure in such cases. Additionally, climate models for Central America consistently predict increases in temperature and decreases in precipitation, particularly during the wet season (Hidalgo et al. 2013; Imbach et al. 2018).

Increased intensity of El Niño-Southern Oscillation events is likely to escalate the frequency of severe droughts for both northern Caribbean and Pacific coasts (Cai et al. 2014; Maurer et al. 2009), which in turn can reduce the supply of sediment to estuarine mangroves and change their salinity. Increases in salinity may reduce tree height, and decreases in rainfall also result in water stress for mangroves, and can affect rates of litter production, and therefore the accumulation and tidal export of organic material (Upton et al. 2018). In contrast, during La Niña years along the southern Caribbean coast, increases in precipitation (Cai et al. 2014; Maurer et al. 2009) will potentially increase runoff and thus sediment supply. Extreme increases in sediment supply have been documented to suffocate mangrove root systems, which interrupts gas exchange causing mass mortality, although more moderate rates may provide enough sediment to support some adaptation to sea level rise (Nardin et al. 2021). The precise ecophysiological responses of mangroves to the range of expected environmental changes remains uncertain. Moreover, global mangrove root production has been reported to average 3.0 ± 0.8 Mg C ha^− 1^ yr^− 1^ broadly equivalent to a productive tropical forest ecosystem, and therefore a major control over Blue Carbon sink capacity (Arnaud et al. 2023). As with litterfall, fine root production and decomposition rates are highly sensitive to climate change impacts including warming and drought (Zeng et al. 2021). Collectively the vulnerability of these processes to climate change impacts can result in reduced carbon sink capacity, and limited ability of mangroves to adapt to sea level rise.

### Future Directions for Blue Carbon Research in Central America

Despite growing interest and a long history of research in the region, our understanding of fundamental processes and the impact of environmental change remains limited. We have identified a series of key questions regarding the processes that regulate the Central American mangrove Blue Carbon sink, assessing how it may respond to future global changes, and how policy and management decision making can build resilience and be effectively financed. We highlight what information is already available to address them, and what else is needed.

#### How do we Improve Estimates of Mangrove Location, Extent, and Carbon Stocks

Informed policy and management decision making relies on up-to-date data on mangrove distribution, carbon storage, and dynamics, including degradation. Refined mapping approaches, supported by the emergence of new satellite data, are increasingly available and adopted, and can provide increased opportunities for more precise mapping, estimates of carbon stocks, and monitoring for conservation and restoration purposes (Friess 2023; Stovall et al. 2021). For example, new data from the highly anticipated BIOMASS and NISAR satellite missions launched in 2024 and 2025 respectively, promise to revolutionise our understanding of Earth’s ecosystems. BIOMASS, employing P-band radar (60 cm wavelength), and NASA-ISRO Synthetic Aperture Radar (NISAR), using L-band radar (30 cm wavelength), are set to provide highly accurate measurements of above-ground biomass, useful in quantifying the extent of and carbon stored in mangrove above ground biomass worldwide. Additionally, the Surface Water and Ocean Topography (SWOT) mission, a collaborative effort by NASA and CNES (The French Space Agency), launched in December 2022, represents a significant advancement in satellite altimetry. SWOT is designed to map ocean surface topography and monitor changes in terrestrial water bodies over time. The data obtained from SWOT will be pivotal in understanding the shifting water levels in river deltas, which are crucial habitats for many mangrove species, thereby offering insights into the impacts of climate change on these vital ecosystems.

Remote sensing and ground-based LiDAR data, however, must be underpinned with the collection of new on-the-ground data, particularly in terms of below-ground biomass and soil carbon storage, to validate such approaches and calibrate them at local and regional scales. Strengthening ongoing national forest inventories (NFI) mapping exercises and field campaigns, using standardised methodologies, will be useful to answer the question of the changes in geographic location, extent, and status of mangrove ecosystems (e.g. assessment of degradation and restoration status). Improved ecological monitoring protocols (for example (SINAC-UNA 2020) for Costa Rica’s protocol) in the long-term, and integration of these and NFI into REDD + measurement, reporting, and verification (MRV) standards would further ensure carbon stock measurements are done systematically, under standardised methodologies and used to feed national-scale reporting. Any additional carbon inventories should be matched to the NFI national sampling grid, echoing previous recommendations (Cifuentes-Jara et al. 2018). It is also critical to unlock existing data within these countries not yet added to global databases. Many times, mangrove data is gathered for specific purposes at the governmental, academic, or local scale. For example, data in Fig. 1 is a representation of what data is available through published literature and a global database, but we recognize that not all data is submitted through these more traditional academic pathways. Access to more grey literature such as management reports, and the integration of those data into policy and inventories, will continuously improve national-level carbon estimates through inter-organizational communication.

#### What are the Responses of Mangrove Ecosystem Dynamics to Environmental Change?

The ecophysiological processes that regulate carbon sequestration will likely be affected by various types of environmental change (see ***Mangroves and anthropogenic change***), which in turn will affect the long-term viability of the Central American Blue Carbon sink. Quantifying future long-term carbon storage requires an understanding of how environment modifies carbon inputs, how biogeochemical processes regulate sequestration rates, how El Niño and climate change impact mangrove survival, and how mortality and recruitment processes differ between species. Measurements of mangrove carbon inputs (e.g. root turnover and litter inputs) remain comparatively limited in Central America, as do measures of carbon fluxes (e.g. carbon dioxide and methane dynamics), lateral carbon flows, and decomposition rates. There is also a need to establish how controls over key ecological processes vary spatially (e.g. between Atlantic and Pacific coast mangroves), temporally (dry season versus wet season), and across geomorphological settings and soil types, given that different areas not only host forests with different carbon dynamics, but also face contrasting risks and differing intensities. Feedback processes are possible, for example with increased atmospheric CO_2_ concentrations enhancing growth, and increasing rates of carbon inputs into sediments (Malhi 2012). To account for this variability, existing protocols for monitoring the processes that regulate carbon flows, alongside new tools and models, will likely be required to integrate relevant environmental and ecological processes (for example measurements of net primary and gross primary productivity), which operate at different scales (Farmer et al. 2011).

While environmental change will impact the ecological processes that regulate sequestration, existing above- and below-ground carbon stocks will also be impacted. Caribbean mangroves are vulnerable to sea level rise due to limited upland space that can be colonised (Alongi 2015). Eutrophication reduces the resilience of mangroves to climate stresses (Simpson et al. 2021). Hurricane events (Taillie et al. 2020), and potentially seismic activity, can cause subsidence and collapse of mangrove carbon stocks (Cahoon et al. 2003; McCloskey and Liu 2013). For example, increased damage severity was observed in Caribbean mangroves during the 2017 hurricane season (amongst the most damaging and costly hurricane seasons on record) compared to the previous eight seasons, with much of the damage persisting throughout the post-hurricane season often due to flooding (Taillie et al. 2020). The increasing intensity and frequency of such events may impact resilience. Carbon stored within mangrove sediments can also be released with increases in temperatures (Arnaud et al. 2023). There is a need to improve monitoring to quantify how stocks will be impacted by such changes at regional and local scales in Central America. For example, there are likely to be significant opportunities from the use of advancing remote sensing technologies on mangrove extent and subsequent carbon stock estimates will be key to ensure that estimates remain consistent in the long-term, and through the integration of various modelling processes.

#### What are the Opportunities for Wise and Sustainable Management, and Conservation and Restoration to Increase Resilience?

Mangroves are widely considered to be amongst the most threatened ecosystems globally (Rull 2022). There have been a number of calls to increase the protection of mangroves and other wetland ecosystems in the region (e.g. Panama; Hoyos-Santillan 2023). Depending on jurisdiction, mangroves are protected by law, potentially influencing and reducing the impacts of some anthropogenic activities. For example in Costa Rica, Guatemala, and Honduras, mangroves are the property of the state and removal, cutting, and land use change are prohibited (Canty et al. 2018). Within the region many mangroves also fall within the boundaries of protected area networks or within Ramsar sites, but may not have specific strategies or plans for their management or protection (Canty et al. 2018). Embedding resilience into mangrove and wetland management first relies on a detailed understanding of mangrove responses to global change. Various strategies are already available, for example the establishment of buffer zones to prevent inland areas from future development and thus allow migration (Samper-Villarreal et al. 2012). Continuous creation and implementation of this type of adaptive management will be required to increase resilience. For example, remote sensing creates opportunities for predicting potential future mangrove habitats. This can inform the design of protected areas, and identify priority areas for conservation and restoration efforts, targeting areas as a priority that provide multiple ecosystem services (Hernández-Blanco et al. 2022).

The long history of pre-Colombian low intensity anthropogenic activity in mangrove ecosystems underlines that mangrove use can be sustainable, but questions remain regarding how such use by local and Indigenous communities may best be supported today especially if coastal communities are growing. The concept of wise and sustainable use of mangroves globally (Gerona-Daga and Salmo 2022), and for other tropical wetland ecosystems more broadly, is increasingly being recognised (Girkin et al. 2023; Wijedasa et al. 2017) with the implication that there are similar opportunities for the management of mangrove ecosystems with respect to provisioning multiple ecosystem services within Central America. Encouraging an improved understanding of traditional knowledge, alongside integrating environmental studies and socio-economic assessments of mangroves, can help inform decision making at local to national levels (Albuquerque et al. 2021; Grimm et al. 2024). In many cases, special use permits or local concessions for sustainable extraction of mangrove resources (e.g. clams) are in place and supported by management plans and biological monitoring. These mechanisms are valuable because it is now widely known that local stewardship of land and resources enhance ecosystem stability while bringing local socioeconomic and resilience benefits (Hagger et al. 2022).

The inclusion of mangroves within national protected area networks and NDCs (Table 1) offers the potential for prioritising protection and restoration of mangroves, as well as opportunities for sustainable use of these ecosystems for food security, local livelihoods, and stronger local stewardship within the region. Most Central American countries have national level targets for mangrove (or wetland management) more broadly included within their NDCs (Girkin and Davidson 2024). The effectiveness of protected areas for underpinning sustainable mangrove management, however, remains unclear given that many protected areas have been reported as having significant shortfalls in resources which hinder effective management (Gill et al. 2017) or lack of management plan implementation. Global Mangrove Alliance national chapters are forming in Central American countries (for example Belize and Panama) helping to bring together national and regional technical experts, policymakers, funding agencies, businesses, and conservation organisations to support better conservation, restoration, and management outcomes for mangroves (Mangrove Alliance 2023). A regional strategy for mangrove management, restoration, and conservation for the Mesoamerican reef region for 2020–2025 has been developed and implemented (Rivas et al. 2020), and a strategy for 2025–2030 will be developed in the near future, which collectively may support better coordination to achieve conservation and restoration targets.

#### How are Countries Using Blue Carbon Finance to Support Wise and Sustainable Use?

Globally, approximately 20% of mangrove forests can already qualify for Blue Carbon financing (Zeng et al. 2021). Within Central America there is an estimated annual mitigation potential of 169,000 t CO_2_e, with an associated VCM value of US$4.79 million (Table 2) in mangrove areas defined as financially viable. Belize is one of the first countries to implement a ‘Blue Bond’ which will unlock ~ US$180 million in conservation finance, including the development of a regulatory framework for Blue Carbon projects. Annually there is an expected US$4 million to finance the protection of resources, with other funds being directed into conservation programs and projects. A Project Finance for Permanence in Belize is aiming to support the improved management of 13 coastal protected areas, 21 marine protected areas, plus mangrove and other coastal ecosystems outside of the formal protected area network, underpinning the restoration of degraded coastal areas, and supporting improvement of local community livelihoods. Costa Rica is working to clarify the structure and functioning of a community-based benefit sharing mechanism that would constitute the first implementation component of that country’s next generation of payment for ecosystem services system focusing on restoring and protecting coastal marine ecosystems.

There is an opportunity for wider adoption of finance mechanisms in the region to support wise and sustainable use, conservation, and restoration. More detailed mapping products (e.g. on local to regional restoration potential) and unified monitoring protocols are also essential for this purpose, to examine current and emergent threats and pressures, and to ensure that restored ecosystems receive due protection (Worthington and Spalding 2018).

The emergence of the voluntary carbon markets, and the sale of carbon credits aligned to high-quality principles (UNFCCC 2022), may create opportunities to provide funding for sustainable use of mangroves, but also may have significant impact on local and Indigenous communities by influencing the activities allowed within given ecosystems (Cisneros-Montemayor et al. 2021). Moreover, such communities may not necessarily receive the benefits of these schemes (Girkin et al. 2022). The Central American region is also home to non-Indigenous minorities, with long-term Afro-Caribbean populations established throughout the region (e.g. Garifuna living on the Honduran north coast for over 200 years) (Mollett 2014) who often rely on mangrove and mangrove-adjacent ecosystems for the provision of ecosystem services, but are frequently marginalised by decision makers (Brondo and Bown 2011). Land titling, namely transferring land titles to Indigenous communities and long-term residents to replace customary land tenure arrangements may provide one route to more effective restoration and conservation outcomes (Lovelock and Brown 2019), particularly given the often complex nature of governance regimes (Recio et al. 2016).

## Conclusions

Mangroves are a crucial component of the Central American Blue Carbon pool but are under threat from a wide range of environmental changes, including the impacts of sea level rise, changes in coastal management, and climate warming. Beyond carbon, Central American mangroves also ensure the continued provision of a wider range of ecosystem services, particularly for local and Indigenous communities who rely on these ecosystems. Positive and equitable outcomes require leadership and engagement at local to national levels to achieve multiple goals at various levels (community, national, regional and global), particularly if the potential of Central American mangroves as a nature-based solution to climate change is to be fully realised. This must be underpinned by continuing to build our knowledge of the changes in distribution and dynamics of mangrove ecosystems, quantifying the spatial and temporal variability of processes in order to create future projects supporting wise and sustainable use, and focusing on applied projects of conservation and restoration in order to achieve regional NDC targets.

## Data Availability

Data sharing is not applicable to this article as no new data were created or analysed in this study. Soil organic carbon stock observations displayed in Fig. 1 were obtained from the Coastal Carbon Atlas accessible at: https://shiny.si.edu/coastal_carbon_atlas.
